# A Biflavonoid-Rich Extract from *Selaginella doederleinii* Hieron. against Throat Carcinoma via Akt/Bad and IKKβ/NF-κB/COX-2 Pathways

**DOI:** 10.3390/ph15121505

**Published:** 2022-12-02

**Authors:** Sisi Wang, Dingrong Wan, Wenqi Liu, Xinyi Kang, Xiuteng Zhou, Fatemeh Sefidkon, Mohaddesehossadat Mahmoud Zadeh Hosseini, Ting Zhang, Xin Pan, Xinzhou Yang

**Affiliations:** 1 School of Pharmaceutical Sciences, South-Central Minzu University, 182 Min-Zu Road, Wuhan 430074, China; 2 State Key Laboratory of Dao-di Herbs, National Resource Center for Chinese Materia Medica, China Academy of Chinese Medical Sciences, Beijing 100700, China; 3 Research Division of Medicinal Plants, Research Institute of Forests and Rangelands, Agricultural Research Education and Extension Organization (AREEO), Tehran P.O. Box 13185-116, Iran; 4 College of Plant Science and Technology, Huazhong Agricultural University, Wuhan 430070, China

**Keywords:** *Selaginella doederleinii*, biflavonoid-rich extraction, throat cancer, apoptosis, NF-κB, Akt

## Abstract

*Selaginella doederleinii* Hieron. is a common pharmacological plant, and this folk herbal medicine and its complex preparations have been widely used for the treatment of throat carcinoma (TC) and several associated complications in traditional Chinese medicine. This study was aimed at investigating the specific anti-throat carcinoma impacts and potential mechanisms of a biflavonoid-rich extract from *S. doederleinii* (SD-BFRE). The phytochemical profiling of SD-BFRE was performed by HPLC-ESI-QTOF-MS and UPLC-PDA, and the detailed pharmacological effects and mechanisms were respectively evaluated in vitro and in vivo. MTT assay, the Transwell assay and flow cytometry were performed to evaluate the abilities of SD-BFRE on inhibiting cell infiltrative growth in TC cells (Hep-2 and FaDu) in in vitro experiments. In vivo experiments used Hep-2 tumor-bearing nude mice to evaluate the anti-TC effect of SD-BFRE. Western blotting was used to explore the potential apoptotic pathway of TC cells. Here, we found that SD-BFRE exhibited anti-proliferation and pro-apoptotic effects in TC cells. Mechanistic studies have identified that SD-BFRE can suppress the activity of IKKβ and IκB-α kinase and then down-regulate the effector proteins of NF-κB/COX-2 signaling. Moreover, SD-BFRE induced apoptosis partly by regulating the Akt/Bad/caspase signaling pathway. Taken together, this study firstly demonstrated that SD-BFRE exerted its anti-TC effects by way of IKKβ/NF-κB/COX-2 and Akt/Bad pathways and might represent a potential chemotherapeutic agent for throat carcinoma.

## 1. Introduction

Throat cancer (TC) is a prevalent refractory and palindromic head and neck squamous cell cancer (HNSCC) with a comparatively high mortality ratio [1,2]. In 2021, the mortality rate among new patients of TC in America was 24.57% [3]. The throat is an organ located in the upper aerodigestive tract that is connected with numerous essential functions including swallowing, breathing and speaking. Thus, both throat carcinoma and its clinical treatment could greatly affect the physical and mental health of patients [4]. In the past, traditional treatments for throat cancer have been dedicated to improving survival rate while retaining the capacity of this organ [2]. For patients with advanced throat cancer, the radiation therapy and chemotherapy have been used as an effective method as well as a prospective organ preservation approach [5]. Recently, notable innovations in genomics and carcinoma immunology have promoted that cancer vaccination could become a personalized carcinoma treatment method. However, HNSCC has a relatively high mutation burden across all cancer types, so it is difficult to efficiently deliver the vaccine [6,7]. Hence, to alleviate the side effects and raise the survival rate of patients, finding more effective anti-throat cancer drugs and developing more personalized and promising strategies are urgently required.

Traditional Chinese medicine (TCM) has a long history to counteract cancer based on a unique treatment system with few side effects, and it has been drawing more and more attention for its use as an anti-tumor strategy [8,9]. In ancient China, Chinese medical scientists believe that heat toxicity is one of the main causes of cancer, which results in the imbalance of Yin–Yang in patients’ bodies [10]. Patients usually experience the following symptoms: localized inflammation and lump, fever and dry mouth [11]. Specifically, ancient Chinese physicians found that herbs with cold properties or traditional prescriptions with a bitter taste significantly ameliorated the progression of cancer based on a unique theory system [12], because those medical herbs alleviate inter heat and reinforce Yin and then achieve the balanced state of “survival with tumor” or “tumor removal and recovery” [13].

*S. doederleinii* Hieron. belongs to the family Selaginellaceae (Pteridophyta), also known as the grass of immortality, which is widely distributed in the Guangxi province of China [14]. Traditional Chinese medical records mentioned that *S. doederleinii* tastes bitter and has cold properties. *S. doederleinii* possesses several unique functions, such as sterilization, detoxication, detumescence and anti-cancer activities, as detailed in the records of the China pharmacopoeia [15]. In Guangxi folk hospitals, this TCM and its complex preparations have been used for the treatment of throat cancer and several associated complications and have shown good clinical effects in throat carcinoma patients [15]. As a result, it has significant advantages in high-efficiency and few side effects in clinical treatment [16]. There are many studies focused on its antitumor activities, such as nasopharyngeal carcinoma [17] and lung carcinoma [18]. However, the detailed mechanisms underlying its anti-throat cancer activity remain unclear.

In the present study, cytotoxicity experiments preliminarily demonstrate that SD-BFRE possesses the best cytotoxicity activity over other fractions (Figure S1). This result is consistent with the literature we reviewed [19,20]. Then, we further examined the potential anti-throat cancer activity of SD-BFRE in vitro and in vivo and explored the possible mechanisms of the function of SD-BFRE to counteract TC.

## 2. Results

### 2.1. UPLC-Q-TOF-MS Analysis of SD-BFRE

**Figure 1:**
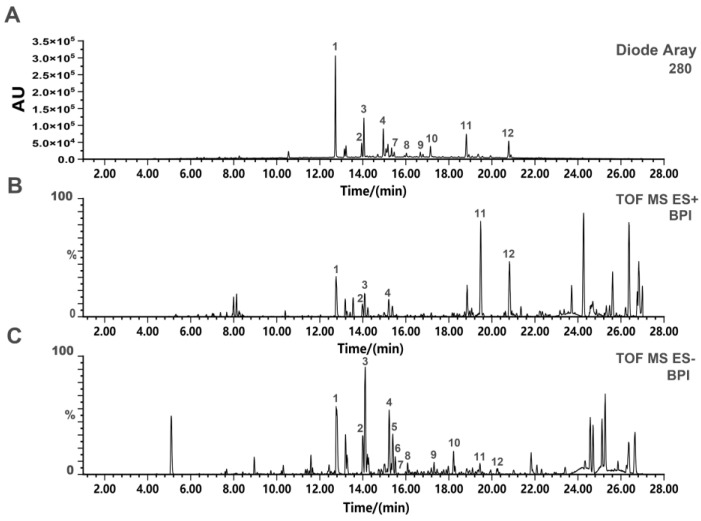
UPLC-Q-TOF-MS analysis of SD-BFRE. (**A**) The basic peak intensity (BPI) chromatograms of SD-BFRE from UPLC-QTOF-MS analysis in the ultraviolet mode. (**B**) Chromatograms in the positive ion mode of SD-BFRE. (**C**) Chromatograms in the negative ion mode of SD-BFRE.

UPLC-Q-TOF-MS method was applicable to rapidly identify the chemical constituents of SD-BFRE. The base peak intensity (BPI) chromatograms of SD-BFRE in both the positive and negative ion modes are displayed in Figure 1B,C. Since flavonoids contain multiple phenolic hydroxyl groups, they have high ionization efficiency in anion mode. Therefore, anion mode is selected and its component structure is speculated on based on relevant literature [14,21]. Based on available literature data and the obtained MS data, a total of 12 biflavonoids were identified [14]. Their structures, the MS data and corresponding retention times of the identified compounds are presented in Table 1 and Figure S2. Moreover, their peak area integration was quantitatively detected by the UPLC-PDA analysis. The results showed that the predominantly identified compounds accounted for more than 85% (Table S1).

### 2.2. SD-BFRE Inhibits Cell Viability and Alters Cell Morphology in TC Cells

**Figure 2:**
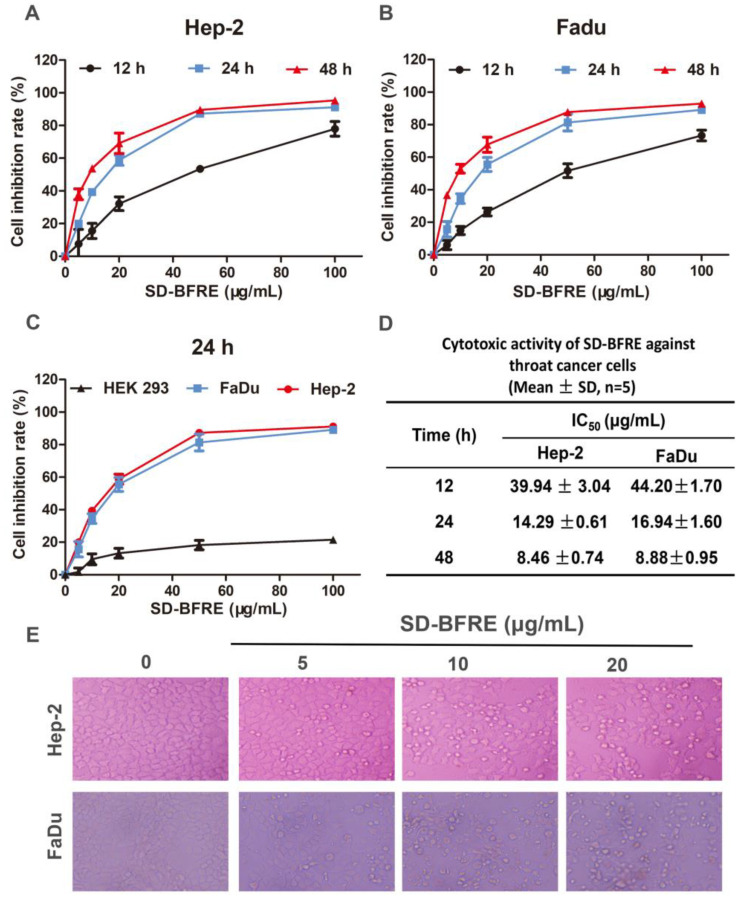
SD-BFRE inhibits cell viability and alters cell morphology in TC cells. (**A**,**B**) Cell inhibition rate of Hep-2 and FaDu cells after they were respectively incubated at different concentration gradients of SD-BFRE for 12 h, 24 h or 48 h. (**C**,**D**) Cell viability and IC_50_ of Hep-2, FaDu and HEK 293 cells after they were respectively treated with different concentration gradients of SD-BFRE for 24 h. (**E**) Morphological observation of two TC cells lines after the treatment of SD-BFRE for 24 h.

SD-BFRE can inhibit human throat cancer cell viability. We used a MTT assay to quantitatively analyze the activities of SD-BFRE on cell proliferation in Hep-2, FaDu and HEK 293 cells. As shown in Figure 2A,B, SD-BFRE dose- and time-dependently inhibited the proliferation of TC cells. It was shown that the IC_50_ of Hep-2 and FaDu cells after 24 h of treatment with SD-BFRE were 14.29 ± 0.61 μg/mL and 16.94 ± 1.60 μg/mL (Figure 2D). Interestingly, SD-BFRE showed less cytotoxicity at high concentrations in the normal embryonic kidney cell line HEK 293 (Figure 2C). Then, we observed the cellular morphological changes and proliferation of TC cells. Plasma membrane blebs and shrunken cells were directly observed after incubation with SD-BFRE (Figure 2E). The results illustrated that SD-BFRE dose- and time-dependently attenuated the growth of throat cancer cells and had little cytotoxicity to normal cell lines.

### 2.3. Effects of SD-BFRE on Cell Migration

**Figure 3:**
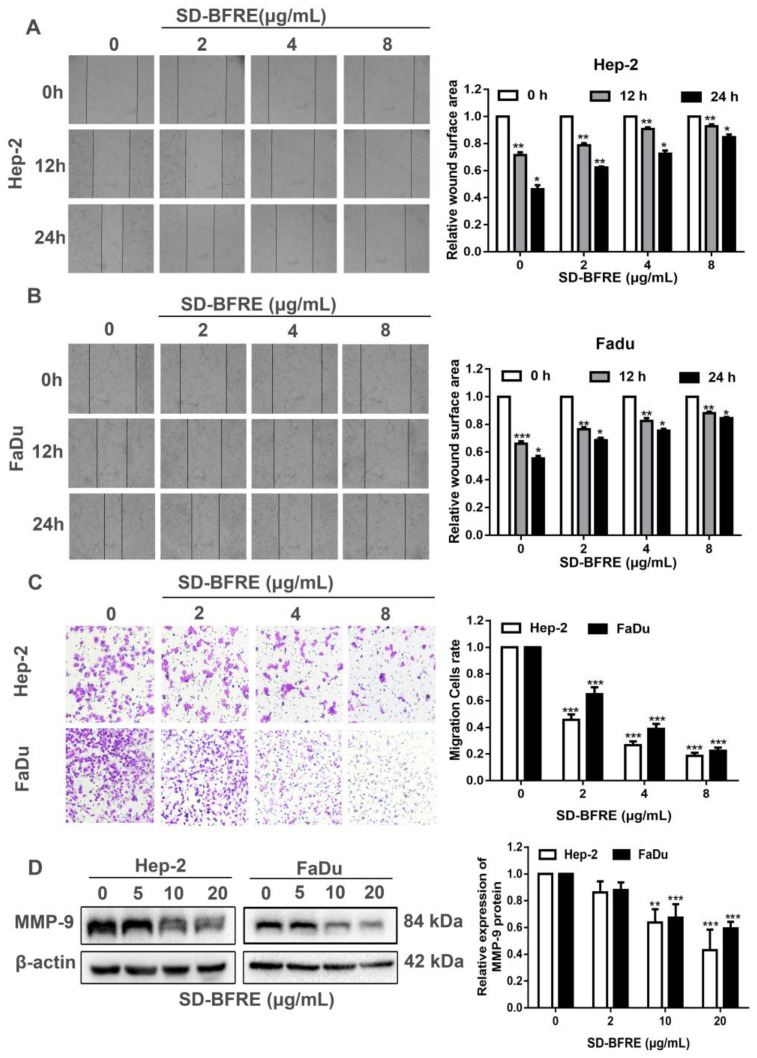
Effects of SD-BFRE on cell migration. (**A**,**B**) Micrographs of Hep-2 and FaDu cells after respective incubation with SD-BFRE (0, 2, 4 and 8 μg/mL), and the wound surface area was counted by Image J software. (**C**) TC cells were seeded in Transwell chambers treated with SD-BFRE. The invading cells were calculated by a fluorescence microscope at the magnification of 200×. (**D**) Effect of SD-BFRE on MMP-9 protein was performed by Western blot assay. β-actin was served as an internal reference (* *p* < 0.05, ** *p* < 0.01 or *** *p* < 0.001, compare to 0 μg/mL).

The Transwell assays and wound healing assays were performed to evaluate the abilities of SD-BFRE at inhibiting cell infiltrative growth in TC cells. The cell viability of two cell lines was obviously attenuated while the SD-BFRE dosage was more than 20 μg/mL. Hence, the concentrations of SD-BFRE were set at 0, 2, 4 and 8 μg/mL. As shown in Figure 3A–C, EC cells obviously inhibited the migration by SD-BFRE, and the effect is concentration related. To test this further, we investigated the expression level of matrix metalloproteinase-9 (MMP-9), which plays a significant role in cell migration; we found that SD-BFRE obviously inhibited the protein level of MMP-9 (Figure 3D). Consequently, SD-BFRE can partly suppress the cell infiltrative growth of TC cells.

### 2.4. SD-BFRE Induces Apoptosis in TC Cells

**Figure 4:**
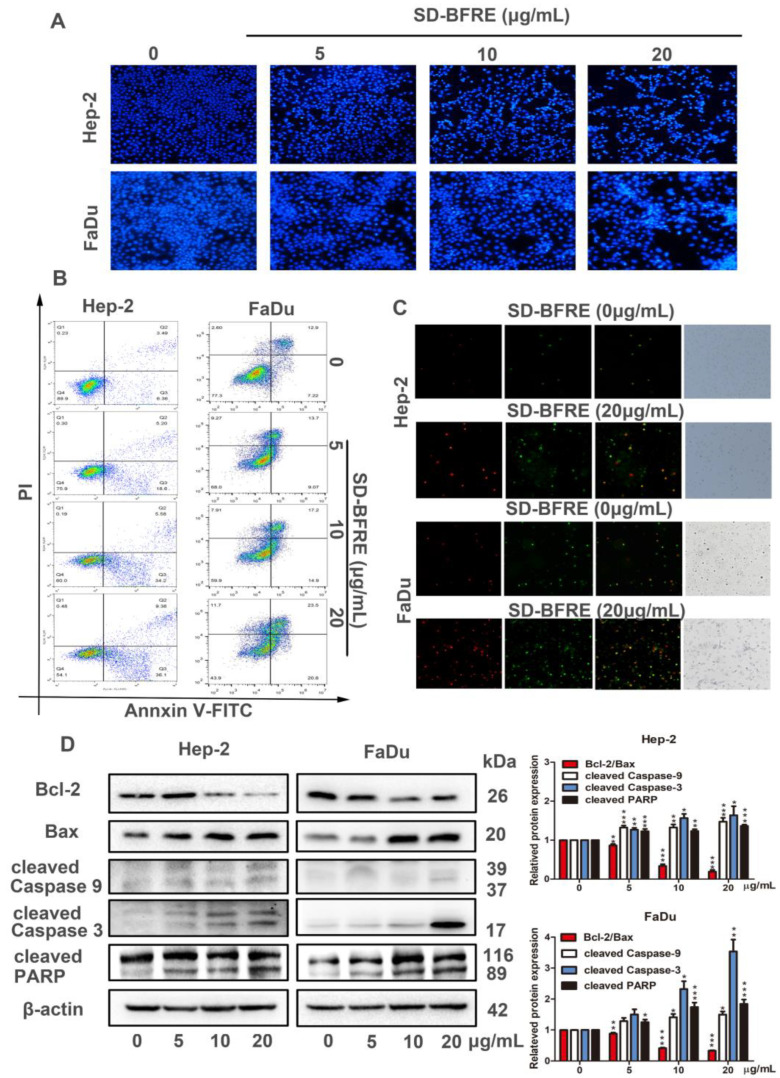
SD-BFRE induces apoptosis in TC cells (**A**) Two TC cells were treated with SD-BFRE for 24 h. Next, the EC cells were stained with Hoechst 33258. Its morphology was observed in a fluorescence microscope. (**B**) Cells were incubated with different concentration gradients of SD-BFRE for 24 h, then stained with Annexin V-FITC and PI. The FACS was applied to detect the proportion of apoptosis. (**C**) Hep-2 and FaDu cells were respectively treated with 0 and 20 μg/mL SD-BFRE, then stained with Annexin V/PI staining and observed in a fluorescence microscope. (**D**) After Hep-2 and FaDu were incubated with SD-BFRE for 24 h, the expressions levels of apoptosis-related proteins were detected by Western blot assay. β-actin was served as an internal reference (* *p* < 0.05, ** *p* < 0.01 or *** *p* < 0.001, compare to 0 μg/mL).

To explore whether SD-BFRE inhibited cell viability was correlated with an increase in apoptosis, Hoechst 33258 staining was served to observe apoptosis in the TC cells. After staining, we can intuitively observe some characteristics of apoptotic cells, such as brilliant blue apoptotic bodies, appeared and the chromatin in the nucleus was condensed. With the increase in SD-BFRE concentration, cells exhibiting these features positively increased (Figure 4A). The flow cytometry combined with a fluorescence microscope were applied to visualize the results of Annexin V-FITC/PI double staining assay. After 24 h incubation with 0, 5, 10 and 20 μg/mL SD-BFRE, the percentages of apoptotic cells (Q2 + Q3) were, respectively, 9.98%, 23.80%, 39.78% and 45.46% in Hep-2 cells and 20.12%, 22.77%, 32.10% and 43.60% in FaDu cells. The result indicated that SD-BFRE dose-dependently induced throat carcinoma cells apoptosis (Figure 4B). Meanwhile, under a fluorescence microscope, the membranes of the viable apoptotic cells were stained with Annexin V-FITC, showing a green fluorescence, and the nucleus of the late apoptotic cells was stained with propidium iodide (PI), producing a red fluorescence. Microscopic observation results directly indicated that SD-BFRE remarkably increased the quantity of apoptotic cells (Figure 4C).

To illustrate the specific mechanism of apoptosis induced by SD-BFRE in throat cancer cell, the expression levels of several apoptosis-related proteins were identified by Western blot analysis in two throat cancer cell lines, including Bcl-2, Bax, Caspase-3/9 and PARP. Compared to the control group, SD-BFRE could obviously upregulate the expression of the Bax, cleave Caspase-3/9 and cleave PARP proteins, but it reduced the expression level of Bcl-2 (Figure 4D). Therefore, SD-BFRE served as a specific and essential mediator, which drives the activation of multiple caspase cascades.

### 2.5. SD-BFRE Inhibited Hep-2 Xenograft Growth and Induced Apoptosis In Vivo

**Figure 5:**
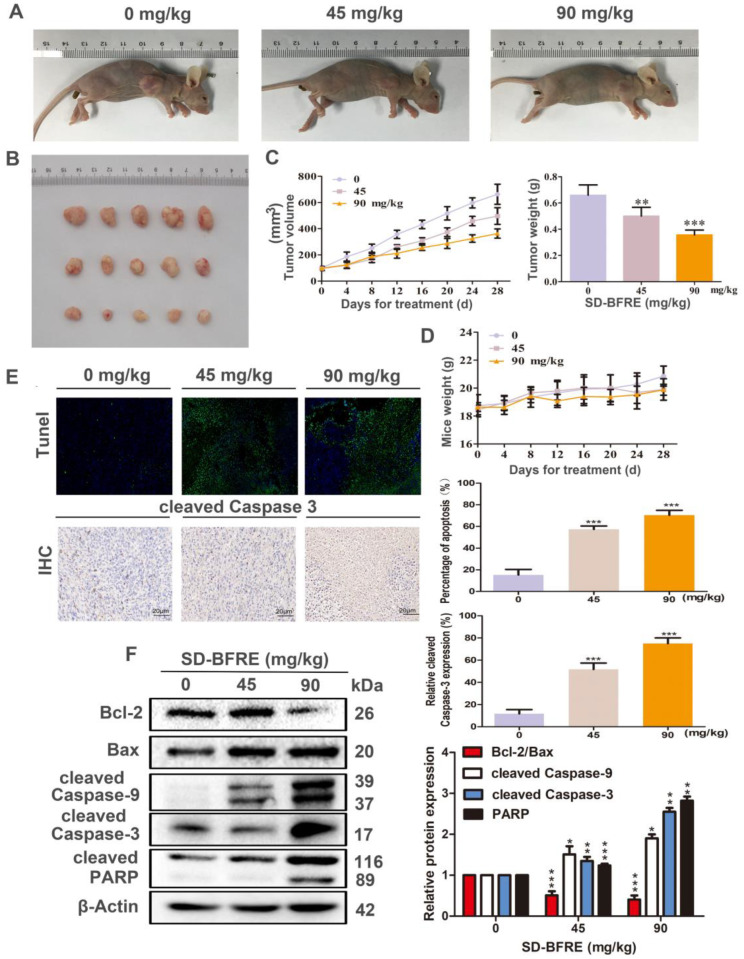
SD-BFRE inhibited Hep-2 xenograft growth and induced apoptosis in vivo. (**A**,**B**) The Hep-2-bearing mice were administrated with different dosage SD-BFRE for 28 days. The size of tumors in treated mice was measured when the experiment was over. (**C**,**D**) Every 4 days during the experiment, the mice were weighed with a balance, the volume of the tumors was measured with a vernier caliper and the tumors were dissected and weighted when the experiment was over. (**E**) Tumor sections of Hep-2 nude mice were subjected to TUNEL and IHC staining. (**F**) Proteins were extracted from SD-BFRE-treated tumor tissues, and the expressions levels of apoptosis-related proteins were detected by Western blot assay. β-actin was served as an internal reference (* *p* < 0.05, ** *p* < 0.01 or *** *p* < 0.001, compared to 0 mg/kg).

Based on the anti-TC cells’ activities of SD-BFRE in vitro, we further explored the effects of SD-BFRE to inhibit tumor growth in vivo by setting up Hep-2-bearing nude mice models. After 28 days of respectively continuous treatment of 0, 45 and 90 mg/kg, the result indicated that SD-BFRE positively decreased both the tumor volumes and weights in treated mice (Figure 5A–C). No significant toxic influences were detected in treated mice (Figure 5D). In addition, the function of SD-BFRE on EC cells’ apoptosis in vivo was visualized by immunohistochemical (IHC) and TUNEL staining. The expression of cleaved-caspase-3 was stained and quantified by IHC, and the nucleus of the apoptotic cells produced green fluorescence by TUNEL. With the increase in the administration of SD-BFRE, the percentage of apoptotic cells in the transplanted tumors gradually rose, as well (Figure 5E). Moreover, the function of SD-BFRE in pro-apoptosis of TC cells in nude mice models was further validated by Western blot assays. It was shown that the apoptosis-related proteins’ variation tendencies were matched with the consequences discovered in vitro (Figure 5F). In summary, these experimental results supported that SD-BFRE can induce throat cancer cells apoptosis in vivo.

### 2.6. SD-BFRE Inhibits NF-κB p65 and COX-2 Signaling In Vitro and In Vivo

**Figure 6:**
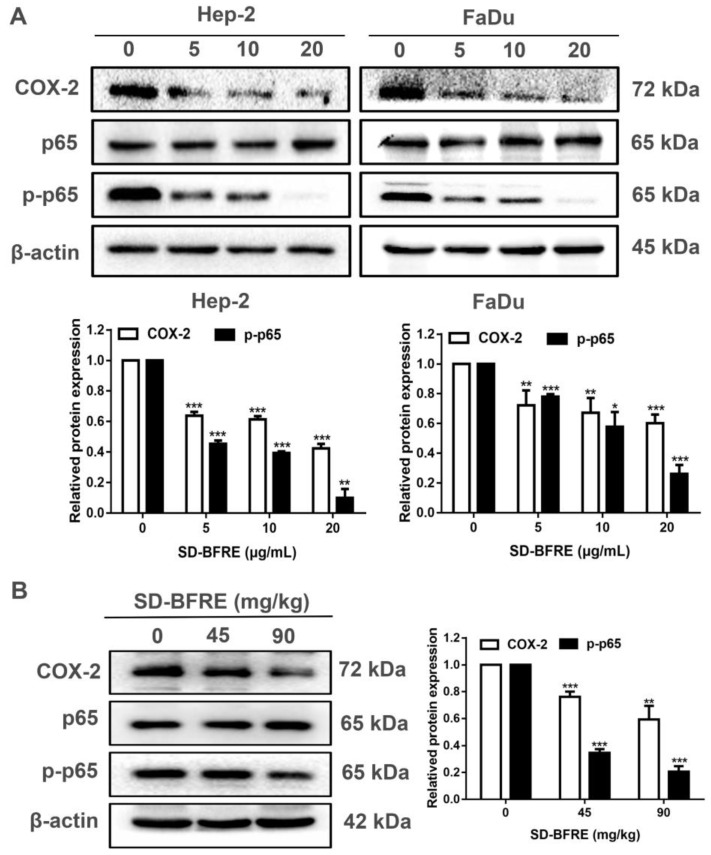
SD-BFRE inhibits NF-κB p65 and COX-2 signaling in vitro and in vivo. (**A**) After being incubated with SD-BFRE for 24 h, the relative expressions of COX-2, NF-κB p65 and p-NF-κB p65 were quantified by Western blot assay. (**B**) Tumor tissue proteins were extracted and prepared, and the relative protein levels of COX-2, NF-κB p65 and p-NF-κB p65 were quantified by Western blot. β-actin was served as an internal reference (* *p* < 0.05, ** *p* < 0.01 or *** *p* < 0.001, compared to 0 μg/mL or 0 mg/kg).

The latest research has indicated that a high expression of Cyclooxygenase-2 (COX-2) is correlated with cell growth and migration of tumor cells [23]. Moreover, the expression of COX-2 was specifically regulated by several transcription factors, including nuclear factor-κB (NF-κB) [24]. The phosphorylation of NF-κB proteins was regarded as an optimal induction of NF-κB target genes, such as p65. To further explore the influence of SD-BFRE on the COX-2/NF-κB signaling pathway, the correlated protein levels were quantified by Western blotting. In vitro, we firstly examined the expression of COX-2, NF-κB p65 and p-NF-κB p65 in two throat carcinoma cell lines, including Hep-2 and FaDu cells. Treatment with SD-BFRE significantly inhibited the protein level of COX-2 and p-NF-κB p65 in Hep-2 and FaDu cells dose-dependently (Figure 6A). In vivo, it was found that the related proteins’ expression trends were consistent with the consequence obtained in vitro (Figure 6A,B). The results suggested that SD-BFRE could attenuate the transcription of NF-κB p-p65 dimer and then suppress the expression of COX-2 in vitro and in vivo.

### 2.7. SD-BFRE Suppresses the NF-κB p65 Transcription by Regulating IKKβ Kinase In Vitro

**Figure 7:**
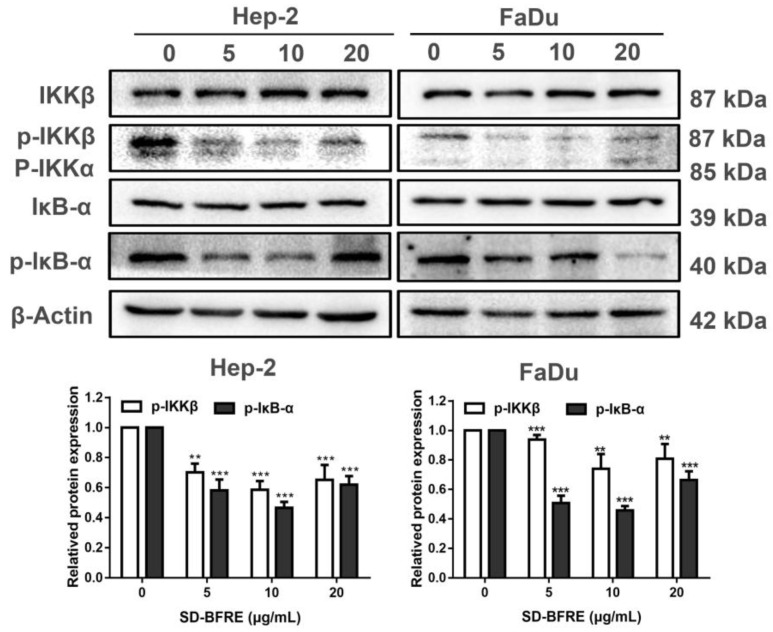
SD-BFRE suppresses NF-κB p65 transcription by regulating IKKβ kinase. (**A**) The relative expressions of p-IKKβ, IKKβ, p-IκBα and IκBα were detected by Western blot assay of TC cells, respectively incubated with SD-BFRE for 24 h. β-actin was served as an internal reference (** *p* < 0.01 or *** *p* < 0.001, compare to 0 μg/mL).

The NF-κB signaling pathway is closely associated with the pathogenesis of inflammation and plays a crucial part in the progression of tumors [25]. NF-κB binds to the inhibitors’ IκB proteins in their resting state and remains inactive in the cytoplasm. Just-activated NF-κB are qualified to translocate to the nucleus and are further involved in gene transcription, nevertheless, the IKK complex is needed for the stimulation of NF-κB [26]. IκB-α is a crucial protein kinase in the canonical NF-κB signaling pathway, and its phosphorylation was majorly regulated by IKKβ. Thus, we investigated the potential mechanisms and evaluated the effect of SD-BFRE on IKK activity in vitro. As shown in Figure 7A, after treatment with SD-BFRE, the proteins’ levels of p-IκB-α and p-IKKβ were gradually decreased in Hep-2 and FaDu cells, while they barely had an impact on the overall protein levels of IKKβ and IκB-α. Therefore, IKKβ kinase might be partly responsible for the inhibition of NF-κB protein activity.

### 2.8. SD-BFRE Induces Apoptosis by Modulating the Akt/Bad Pathway In Vivo and In Vitro

**Figure 8:**
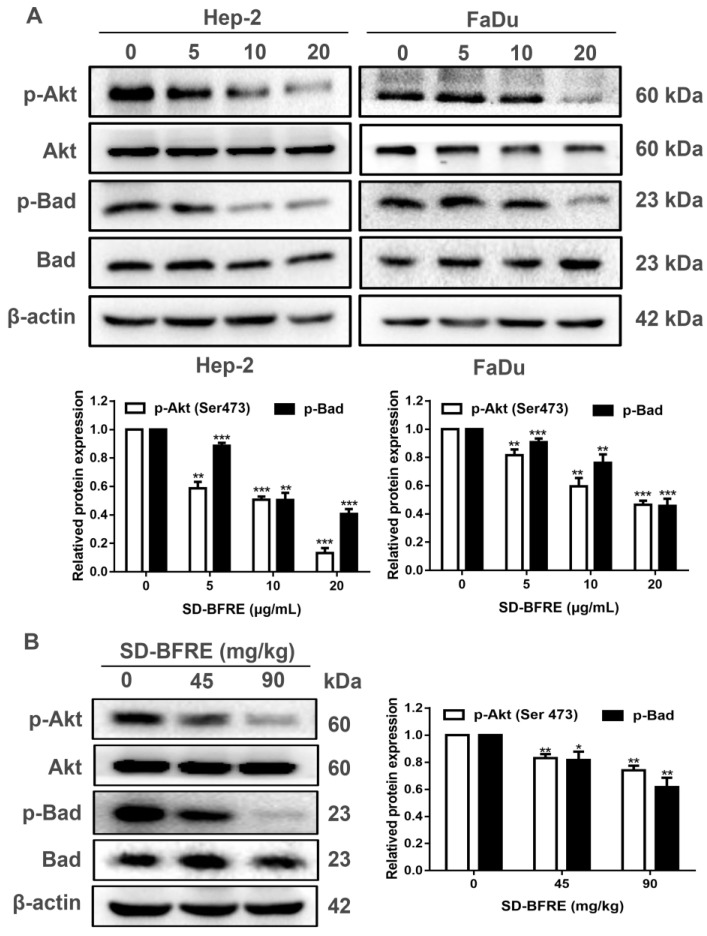
SD-BFRE induces apoptosis by modulating the Akt/Bad pathway in vivo and in vitro. (**A**) The proteins levels of p-Akt, p-Bad, Akt and Bad were detected by Western blot analysis of TC cells after being treated with SD-BFRE (0, 5, 10 and 20 μg/mL). β-actin served as an internal reference. (**B**) Tumor tissue proteins were extracted and prepared, and the relative protein levels of p-Akt, p-Bad, Akt and Bad were detected by Western blot. β-actin served as an internal reference (* *p* < 0.05, ** *p* < 0.01 or *** *p* < 0.001, compare to 0 μg/mL or 0 mg/kg).

In cancer cells, protein kinase B (Akt, PKB) can regulate a variety of signaling pathways, including inhibiting cell apoptosis. Moreover, NF-κB can be regulated by the PI3K/Akt signaling pathway, then eventually translocate to the nucleus where it suppresses tumor cells apoptosis [27]. In order to detect whether SD-BFRE induced apoptosis is related to the PI3K/Akt pathway, in vitro and in vivo Western blot assay was applied to visualize the expression of related proteins. As shown in Figure 8A,B, the result suggests that SD-BFRE down-regulated the expression of p-Akt and p-Bad, but barely inhibited the overall expression levels of Akt and Bad protein in cancer cells. This indicated that SD-BFRE inhibits the proliferation of TC cells in vivo and in vitro through the PI3K/Akt signaling pathway.

## 3. Discussion

Throat carcinoma is the second most refractory and palindromic head and neck squamous cell carcinoma (HNSCC) [2]. Nevertheless, the present treatment of throat cancer makes its prognosis unsatisfactory. The main factors affecting the poor prognosis in throat cancer include: (a) infinite proliferation, (b) infiltrative growth, (c) resistance to apoptosis and (d) inflammation-related carcinogenesis. Thus, it is important to discover novel and promising chemotherapeutic drugs to mitigate these factors. In this study, we discovered that a biflavonoid-rich extract from *S. doederleinii* inhibited throat cell proliferation and then detected the mechanism underlying its anti-TC effects.

Infiltrative growth is one of the most major causes of refractory throat cancer [28]. Both migration and invasion are essential pre-conditions for infiltrative growth. Moreover, the regression of the extracellular matrix is an important aspect. MMP-9 is a gelatinase, which is a crucial member of the MMP family, and MMP-9 is also in charge of the degradation of the extracellular matrix [29]. The result illustrated that SD-BFRE can significantly decrease the expression of MMP-9 (Figure 3D). Furthermore, the Transwell and scratch assay suggested that SD-BFRE can positively inhibit the metastasis and invasion (Figure 3A–C).

Apoptosis is a kind of programmed cell death, which was instrumental in damaging cells and maintaining cellular homeostasis [30]. Presently, most of the clinical anti-tumor drugs work through the apoptosis pathway [30,31]. During the apoptosis process, the early events are cell shrinkage and floating, and then the cells decompose into apoptotic bodies accompanying chromatin condensation and nucleus fragmentation [32]. In this study, Hoechst 33258 staining and Annexin V-FITC/PI assay demonstrated that SD-BFRE can induce the apoptosis of throat carcinoma cells dose-dependently (Figure 4A–C). The intrinsic apoptotic pathway (the mitochondrial pathway) is mediated by the Bcl-2 protein family encompassing anti-apoptotic protein (Bcl-2) and pro-apoptotic protein (Bax) [33]. A decreased ratio of Bcl-2/Bax was used to represent induction of cell apoptosis. The Bcl-2 family could also regulate the release of cytochrome c (Cyt-c) [34]. Under the stimulation of apoptosis-inducing factors, Cyt-c forms apoptotic bodies with Caspase-9 precursors in the cytoplasm, resulting in the activation of cleaved Caspase-9. Subsequently, the activated cleaved Caspase-9 further cleaves the downstream executor Caspase-3 to activate it and eventually induces apoptosis [35]. Caspase-3 can further regulate a downstream protein Poly ADP-ribose polymerase (PARP), and PARP is responsible for the repair of carcinoma cells [36]. In this study, Western blot assay in vitro showed that SD-BFRE can induce apoptosis by activating the mitochondrial pathway in throat carcer cell lines (Figure 4d). Moreover, Hep-2-bearing nude mice models were established to investigate the pro-apoptotic activity of SD-BFRE in vivo. After being treated with SD-BFRE in vivo models, the growth of transplanted tumors was dose-dependently inhibited, and its pro-apoptotic effect was exerted by activating the intrinsic apoptotic pathway (the mitochondrial pathway) in vivo (Figure 5).

Akt (protein kinase B, PKB) is a common target for anti-tumor treatment, which is known as a downstream target protein of PI3K. Research has demonstrated that the activation of Akt is engaged in a variety of signaling pathways [37], including inhibiting the activation of NF-κB and the cell apoptosis [27,38]. PI3K specifically regulates the activity of Akt by promoting the phosphorylation, so the decrease in phosphorylated Akt (p-Akt Ser473) expression is a significant indicator of the activation of the PI3K/Akt pathway. Activated Akt can phosphorylate a variety of substrates, thereby attenuating pro-apoptotic proteins (such as Bad). The results, both in vitro and in vivo, confirmed SD-BFRE-induced apoptosis in throat carcer cells by inhibiting the PI3K/Akt/Bad signaling pathway (Figure 8).

Chronic inflammation is another key cause of cancer, and it has been reported to exacerbate the progression of cancer [39,40]. COX-2 is regarded as a molecular market for inflammatory diseases, its overexpression causing an unsatisfactory prognosis and the malignancy of cancer [41]. Nuclear factor κB (NF-κB) is a cis-acting element of the COX-2 and is associated with the aberrant expression of COX-2 [42]. NF-κB plays a key role in inflammation-related carcinogenesis and acts as a regulator in the occurrence and progression of carcinoma. NF-κB is a Rel family transcription factor, which mainly exists in the form of a heterodimer composed of a p50 and p65 dimer. The target genes are located in the C-terminal sequences of the p65 dimer to activate the transcriptional activity and eventually accelerate cell proliferation, while the p50 dimer does not possess this sequence. Hence, the phosphorylation ratio of NF-κB p65 was used to represent the transcription activity of NF-κB [43,44]. In this study, Western blot analysis consequences indicate that SD-BFRE might inhibit the expression of COX-2 and phosphorylation of NF-κB p65 in a dose-dependent manner (Figure 6). Thus, it demonstrated that SD-BFRE can suppress the nuclear transfer activity of the NF-κB p65 dimer and then inhibit the transcription activity of NF-κB and the expression of COX-2.

The activation of the NF-κB pathway has been identified as a major contributor of inflammation, which is also banding with the overexpression of COX-2. Meanwhile, in the development and progression of cancer, NF-κB can immediately engaged in this process by regulating the expression of genes [45]. In classical NF-κB activating pathways, before activation, NF-κB p65 is combined with an IKK kinase complex, commonly named as IκBα, in cytoplasm [46]. IKKβ is the key catalytic subunit which is responsible for the activation of IκB-α. When stimulated by catalytic subunits (IKKα and IKKβ, IKKg), IκBα is phosphorylated and degraded apart from NF-κB p65 via the ubiquitin–proteasome pathway [47]. Then, the activated NF-κB heterodimer translocated into the nucleus and finally acted as a transcription factor of inflammatory genes [47]. By Western blotting analysis in two throat cancer cell lines, we found that SD-BFRE inhibited IKKβ and IκB-α kinase activity. These findings suggest the transcriptional activity of NF-κB was notably abrogated by SD-BFRE through attenuating the activity of IKKβ (Figure 7A).

**Figure 9:**
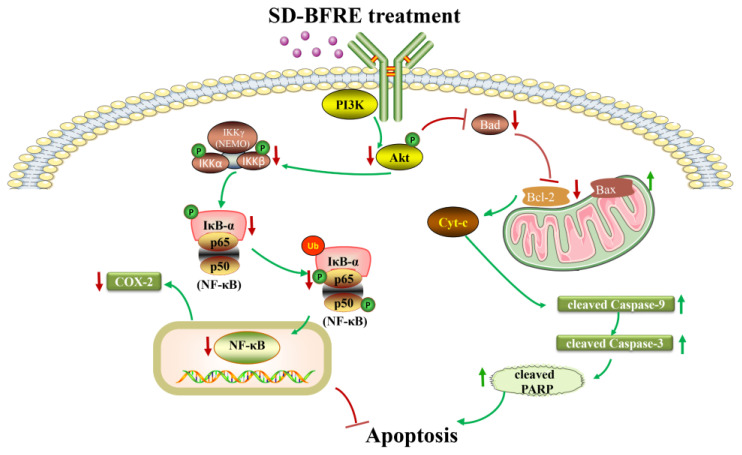
The mechanisms of SD-BFRE induced-apoptosis and anti-proliferation of throat carcinoma cells.

Taken together, SD-BFRE can effectively inhibit throat carcinoma cell proliferation and induce apoptosis by negatively regulating the NF-κB/COX-2 and the Akt/Bad/caspase cascade signaling pathways. Moreover, SD-BFRE suppressed IKKβ and IκB-α kinase activity, hence inhibiting the NF-κB proteins entering the nucleus and interrupting NF-κB binding on the COX-2 promoter, thereby reducing the expression of NF-κB and COX-2 in throat cancer cells. In addition, the phosphorylation of intracellular Akt/Bad signals was down-regulated (Figure 9). These findings provide a possible anticancer mechanism for SD-BFRE treatment. SD-BFRE thus represents a potential chemotherapeutic agent for throat carcinoma.

## 4. Materials and Methods

### 4.1. Chemicals and Reagents

The chemical solvents were analytic reagents, except that the acetonitrile (MeCN) and methanol used in chromatography were of HPLC-grade. FBS and antibiotics (streptomycin and penicillin) were obtained from Solarbio (Beijing, China). Cell culture medium DMEM was procured from Sigma-Aldrich (Shanghai, China). The 24-well Transwell plates were procured from Corning. Antibodies including MMP-9 (CST, #13667, MA, USA), Bax (CST, #5023, MA, USA), Bcl-2 (CST, #15071, MA, USA), PARP (CST, #9542, MA, USA), Caspase-3 (CST, #9662, MA, USA), Caspase-9 (CST, #9508, MA, USA), Akt (CST, #4691, MA, USA), phosopho-Akt (Ser473) (CST, #4060, MA, USA), Bad (CST, #9268, MA, USA), phosopho-Bad (CST, #4366, MA, USA), COX-2 (CST, #4842, MA, USA), NF-κB p65 (CST, #8242, MA, USA), phosopho-NF-κB p65 (CST, #3033, MA, USA), IKKβ (CST, #8943, MA, USA), phosopho-IKKα/β (CST, #2697, MA, USA), phosopho-IκB-α (CST, #9246, MA, USA), IκB-α (CST, #4814, MA, USA) and β-actin (Biopm, PMK081S, Wuhan, China). along with all the matching secondary antibodies, including anti-rabbit IgG (ABclonal, AS029, Wuhan, China) and anti-mouse IgG (ABclonal, AS061, Wuhan, China).

### 4.2. Plant Material

The whole wild dried herbs of *Selaginella doederleinii* Hieron. were procured from an herbal medicine market of Hechi city, Guangxi province, China in June 2019. All the herbs were ascertained by Prof. Dingrong Wan, School of Pharmaceutical Sciences, South-Central University for Nationalities (SCUN), Wuhan, China. The certificate specimen (SC0064) is retained in the School of Pharmacy, SCUN.

### 4.3. Preparation and Chemical Elucidation of SD-BFRE

The whole dried herb of SD (450 g) was freeze-dried, crushed and filtered successively to obtain a uniform powder. Next, the powder was totally macerated five times separately with 95% ethanol. Under the condition of reduced pressure, the solvent was evaporated to obtain an extract (54.7 g). Next, the crude extract was successively partitioned with different concentration gradients of organic solvents to respectively obtain n-BuOH fraction (20.1 g), acetic ether fraction (7.7 g) and PE fraction (3.8 g). The D101 macroporous resin column chromatography (200 g, Sinopharm Chemical Reagent Co., Ltd., Shanghai, China) was applied to separate the acetic ether extract (6 g) with progressively higher concentrations of ethanol to provide eluents. According to the HPLC analysis of chromatogram, we merged the different eluents into six fractions (Frs. A-F). Fr. D indicated the strongest cytotoxicity to counteract two throat carcinoma cell lines as the purified SD-BFRE, a total of 2.5 g.

The UPLC-Q-TOF-MS analysis was applied on an ultra-high-performance liquid chromatography (UPLC) system (Waters Corp., Milford, MA/USA) consisting of a binary pump, an auto-sampler, a degasser and a column compartment with a high throughput G2Si High-definition mass spectrometry (Waters Q-TOF SYNAPT™, Waters Corp, Manchester, England) equipped with an electrospray (ESI) interface. The samples were separated on a Waters ACQUITY UPLC HSS T3 column (100 mm × 2.1 mm, 1.8 μm). The injection volume of the sample was 2 μL, and the flow rate was at 0.4 mL/min. The gradient elution was: 0–25 min, 2–95%. Meanwhile, the temperature of the column was acquired at 40 °C, the detection wavelength was acquired over 200–500 nm and the mass spectrometry (MS) detections applied the following operation parameters: sampling cone, 25 V; capillary voltage, extraction cone, 4 V; 3.0 kV (negative mode); desolvation temperature, 350 °C; source temperature, 120 °C. The desolvation gas was nitrogen, and its flow rates were set at 50 and 900 L/h. Mass spectra were set at m/z 50–1200. The data were processed by Triple TOF™ 5600 (AB SCIEX) and recorded by Analyst TF 1.6 software.

### 4.4. Cell Culture

Hep-2 (laryngeal cancer cell originated from human) was procured from the American Type Culture Collection (ATCC, Manassas, VA, USA). FaDu (hypopharyngeal carcinoma cell originated from human) and the embryonic kidney cell line HEK 293 were procured from the Shanghai Institute of Biochemistry and Cell Biology (Shanghai, China). These cells were maintained in an incubator comprising a constant atmosphere (37 °C) with 5% CO_2_. The formula for the cell culture medium is as follows: 1% antibiotics (penicillin and streptomycin) and 10% FBS in DMEM/MEM.

### 4.5. Cytotoxicity by MTT Assay

Hep-2, FaDu and HEK 293 cells were plated in 1 × 10^4^ cells/well in 96-well plates and cultured until about 80% confluence. Next, cells were treated with five different concentration gradients of SD-BFRE, then respectively incubated for 12, 24 and 48 h. After processing, the medium was removed. Subsequently, 100 μL mixed reagents (10% MTT and 90% DMEM) were added to each well, then it was put in an incubator of 37 °C constant temperature. After 45–80 min, we added 150 μL DMSO to each well. Under the detection wavelength (562 nm), the absorbance was detected by a microplate reader (Bio-Rad, Hercules, CA, USA). The equation for calculating the inhibition rate was as follows:

Inhibition rate (%) = [1 − (ODsample − ODblank)/(ODcontrol − ODblank)] × 100.

The IC_50_ value of SD-BFRE was counted applying the GraphPad Prism 5.0 Software.

### 4.6. Cell Migration Assays

In the wound healing assay, two TC cell lines in the logarithmic phase were seeded at 2 × 10^5^ cells/well in 6-well plates, then cultivated overnight until about 90% fusion. After treatment with different concentration gradients of SD-BFRE (0, 2, 4 and 8 μg/mL), wounds were generated by scraping each plate’s surface with a sterile 200 μL pipette tip held at an upright elevation. After being incubated with SD-BFRE for 0, 12 and 24 h, respectively, a phase inverted microscope (Soptop ICX41, Ningbo, China) was applied to photograph the scratch wound images at a magnification of 40×. Image J software was used to calculate the measurements of the wound’s surface area. Meanwhile, the Transwell assay was performed as described above. The upper chambers of 24-well Transwell plates were contained with Hep-2 and FaDu cells and DMEM (FBS-free), and 500 μL of DMEM with 10% FBS were added into the bottom chambers. When the cell sticks to the wall, both the bottom and top chambers contained the same liquid of SD-BFRE (0, 2, 4 and 8 μg/mL). After incubating in chambers for 24 h, cotton swabs were used to wipe the non-invasive cells on the upper membrane surfaces. After processing, the invading cells were fixed with methyl alcohol and stained with Crystal Violet solution. Next, under a phase inverted microscope (Soptop ICX41, Ningbo, China), the extension of the cell migration images was photographed. The invading cells’ numbers were counted in three random areas. The cell mobility was analyzed by Image J software.

### 4.7. Morphology Observation and Hoechst 33258 Staining

Similar to the cell migration assay, two TC cell lines were inoculated in 6-well plates, then cultivated overnight until about 80−90% fusion. The cells were treated with different concentration gradients of SD-BFRE (0, 5, 10 and 20 μg/mL) for 24 h, and later on, after processing, an inverted phase contrast microscope (Soptop ICX41, Ningbo, China) was used to photograph the changes in cellular morphological at a magnification of 40×. Subsequently, the original component was removed, and cells were fixed with a mixed solution (methanol: glacial acetic acid = 1:3). Then, under dark conditions, the cell was stained with Hoechst 33258 (Beyotime, Shanghai, China). Finally, the excitation wavelength and emission wavelength were respectively set to 350 nm and 460 nm, and a fluorescence microscope (Soptop ICX41, Ningbo, China) was applied to observe and photograph the cellular morphology at a magnification of 40×.

### 4.8. Annexin V-FITC/PI Staining Assay

Consistent with the previous description, throat carcinoma cells were inoculated in 6-well plates, then cultivated overnight until about 80−90% fusion after incubation with different concentration gradients (0, 5, 10 and 20 μg/mL) of SD-BFRE for 24 h. Next, cells were digested with EDTA-free trypsin. The detailed staining procedures were performed under the protocol of Annexin V-FITC/PI staining kit (BestBio, Shanghai, China). Finally, the percentage of apoptotic cells was immediately observed with a fluorescence microscope and quantitatively calculated by FCM (Guava easyCyte, United States). Subsequently, the proportion of SD-BFRE on throat cancer cell apoptotic was analyzed by FlowJo V10 software.

### 4.9. Proteins Extracts and Western Blot Analysis

Throat cancer cells were plated in the medium and allowed to cultivate until about 70−80% fusion. After being treated with four concentration gradients (0, 5, 10 and 20 μg/mL) of SD-BFRE for 24 h, the cells were separated and extracted in a RIPA lysis buffer (Beyotime, Shanghai, China). The supernatant was collected and its concentration was measured by the BCA kit (Beyotime, Shanghai, China). Next, the protein was put in slightly boiling water for 10 minutes, then stored in an −80 °C freezer for further analysis. The collection and extraction of tumor tissue protein was performed as mentioned previously. Equivalent amounts of the protein (40 µg) were parted by SDS-PAGE (10% or 12.5%) and then transferred to PVDF membranes (Bio-Rad, CA, USA) by electrophoresis. The membranes containing bands of protein were blocked with 5% skim milk powder dissolved in TBST for 2 h. After processing, the membranes were incubated with the primary antibody at 4 °C overnight. After being washed three times the next day, the membranes were incubated with the corresponding secondary antibody for 1 h 40 min. The HRP ECL system (Bio-Rad, CA, USA) was applicable to show the protein band. Meanwhile, the variation tendency of protein was analyzed by Image Lab software.

### 4.10. Tumor-Bearing Nude Mice Models and In Vivo Treatment

An experimental tumor model was produced by subcutaneous injection of Hep-2 cells (1 × 10^7^) in the upper right back of each female nude mouse (BALB/c, SPF grade, 17–20 g, 4−5 weeks old). When the tumors approximately reached 100 mm^3^, the nude mice were randomly separated into three groups. The three groups were given the corresponding concentration gradients of SD-BFRE by intraperitoneal injection for four weeks. The transplanted tumor volume was measured every four days. After 28 days, the transplanted tumors were removed and weighted for further analysis. The calculated formula of transplanted tumor volume was as follows:

Tumor volume = 0.5 × width^2^ × length

### 4.11. TUNEL Staining

After the isolated tumor tissues were fixed with 4% methanol (PH 7.4) for 24 h and eluted by gradient (ethanol, water), the tissue samples were completely immersed in paraffin solution for incubation until they were completely embedded to form wax blocks. Then, the paraffin blocks were cut into 5-micron sections with a paraffin pathological slicer. According to the official instructions, TUNEL assay kit (Basel, Switzerland) was used for analysis of each section, and pictures were taken under a microscope at 200×/400× magnification.

### 4.12. Immunohistochemical Examinations of Transplanted Tumor Tissues (IHC)

On the basis of the above steps, briefly, tumors were encapsulated in paraffin. Their slices were selected and cut into 5-micron sections, then, they were incubated with the corresponding primary antibody and visualized by horseradish peroxidase conjugated secondary antibodies. After processing, the sample was photographed at 200×/400× magnification. The staining result was analyzed by Image J software.

### 4.13. Statistical Analysis

One-way analysis of variance (ANOVA) was applicable to analyze the differences between groups. A *p* value < 0.05 was regarded as statistically significant. All data were shown as means ± standard error, which were obtained from three independent experiments. GraphPad Prism 6.0 software was applied to perform statistical analyses.

## 5. Conclusions

In conclusion, the current study demonstrates that SD-BFRE can effectively inhibit throat carcinoma cell proliferation and induce apoptosis both in vivo and in vitro. Mechanistic studies have showed that SD-BFRE exerted its anti-TC effects by negatively regulating the NF-κB/COX-2 and the Akt/Bad/caspase cascade signaling pathways. These findings have confirmed that SD-BFRE represents a potential chemotherapeutic agent for throat carcinoma treatment. Nonetheless, there still exist some limitations that need to be improved in subsequent work. In this study, we did not figure out which biflavonoid plays a key role in SD-BFRE for its anti-throat cancer effects. Therefore, later studies will aim at finding the main active biflavonoid from SD-BFRE for its anti-TC cell effects by in vitro experiment. Then, we can further investigate the in-depth anti-TC mechanisms of this compound and provide a potential strategy for clinical treatment.

## Figures and Tables

**Table 1:** Identification of biflavonoid from SD-BFRE by UPLC-QTOF-MS.

No.	*t_R_*	Molecular	Ion Type	Molecular	Exact Mass	Compound	Reference
	(min)	Iron (m/z)		Formula			
1	12.81	537.0807	[M-H]^−^	C_30_H_18_O_10_	538.0900	Amentoflavone	[14]
2	13.99	538.0969	[M-H]^−^	C_30_H_19_O_10_	539.1056	2,3-Dihydro-3′,3′’’’-biapigenin	[14]
3	14.06	541.1167	[M-H]^−^	C_30_H_22_O_10_	542.1213	2,3,2′’,3′’-Tetrahydroochnaflavone	[22]
4	15.00	539.1029	[M-H]^−^	C_30_H_20_O_10_	540.1055	2,3-dihydroochnaflavone	[22]
5	15.21	537.1248	[M-H]^−^	C_30_H_18_O_10_	538.1369	Delicaflavone	[22]
6	15.22	537.1096	[M-H]^−^	C_30_H_18_O_10_	538.1269	Ochnaflavone	[22]
7	15.37	537.0983	[M-H]^−^	C_30_H_18_O_10_	538.1086	Hinokifllavone	[14]
8	16.06	551.0997	[M-H]^−^	C_31_H_20_O_10_	552.1213	Bilobetin	[14]
9	16.84	565.1285	[M-H]^−^	C_32_H_22_O_10_	566.1365	Ginkgetin	[21]
10	17.18	551.0983	[M-H]^−^	C_31_H_20_O_10_	552.1666	Podocarpusflavone A	[21]
11	18.83	579.1281	[M-H]^−^	C_33_H_24_O_10_	580.1395	Heveaflavone	[14]
12	20.82	593.1569	[M-H]^−^	C_34_H_26_O_10_	594.1526	7,4′,7′’,4′’’-Tetra	[14]
						O-methyl-amentoflavone	

## Data Availability

Data is contained in the main text and supplementary materials.

## Supplementary Materials

The following supporting information can be downloaded at: https://www.mdpi.com/article/10.3390/ph15121505/s1, Figure S1: Cell inhibition rate of Hep-2 and FaDu cells after they were respectively incubated different concentration gradients of SD-BFRE, SD-EA and SDE for 12 h, 24 h or 48 h; Figure S2: The chemical structure of 12 biflavonoids identified from SD-BFRE by UPLC-Q-TOF-MS; Table S1: The peak area integration of biflavonoid from SD-BFRE by UPLC-PDA.

## Abbreviations

HPLC, high-performance liquid chromatography; DMEM, Dulbecco’s modified eagle medium; PBS, Phosphate buffer saline; ECL, Electrochemiluminescence; PI, Propidium iodide; FITC, Fluorescein Isothiocyanate; MTT, 3-(4,5-Dimethylthiazol-2-yl)-2,5-diphenyltetrazolium bromide; SDS-PAGE, Sodium dodecyl sulfate polyacrylamide gel electrophoresis; TBST, Western Blot wash and incubation buffer with tween; BCA, Bicinchoninic acid; PVDF, Polyvinylidene fluoride; TUNEL, Terminal deoxynucleotidyl transferase-mediated dUTP-biotin nick end labeling assay.
