# Supplementary material for: A Biflavonoid-Rich Extract from Selaginella doederleinii Hieron. against Throat Carcinoma via Akt/Bad and IKKβ/NF-κB/COX-2 Pathways
Source: Pharmaceuticals (Basel). 2022 Dec 2;15(12):1505. doi: 10.3390/ph15121505 (PMC9785591; doi:10.3390/ph15121505)
Supplement: Supplementary file 1 [file pharmaceuticals-15-01505-s001.zip › Figure S1.pdf]

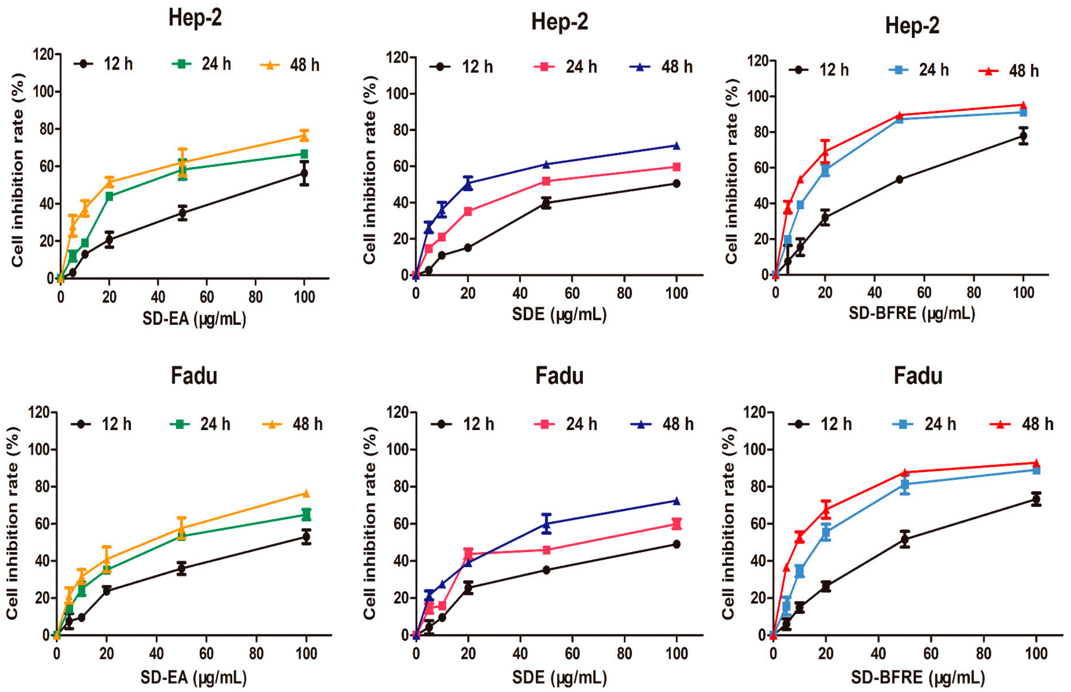

Cytotoxic activity of SD-BFRE,SD-EA,SDE against Hep-2 (Mean±SD,n=3)

Times (h)	IC_{50} (µg/mL)		
	SD-BFRE	SD-EA	SDE
12	39.94 ±3.04	80.94 ± 7.30	89.03 ±5.15
24	14.29 ±0.61	36.16 ± 2.60	43.82 ± 3.11
48	8.46 ±0.74	20.08 ± 2.01	28.48 ±4.83

Cytotoxic activity of SD-BFRE,SD-EA,SDE against FaDu (Mean±SD,n=3)

Times (h)	IC_{50} (µg/mL)		
	SD-BFRE	SD-EA	SDE
12	44.20 ±1.70	89.03 ± 5.15	97.30 ±1.95
24	16.94 ±1.60	43.82 ± 3.11	53.68 ± 3.37
48	8.88 ±0.95	28.08 ± 4.83	33.14 ±3.81

Figure S1: Cell inhibition rate of Hep-2 and FaDu cells after they were respectively incubated different concentration gradients of SD-BFRE, SD-EA, SDE for 12 h, 24 h or 48 h.
