# Supplementary material for: A Biflavonoid-Rich Extract from Selaginella doederleinii Hieron. against Throat Carcinoma via Akt/Bad and IKKβ/NF-κB/COX-2 Pathways
Source: Pharmaceuticals (Basel). 2022 Dec 2;15(12):1505. doi: 10.3390/ph15121505 (PMC9785591; doi:10.3390/ph15121505)
Supplement: Supplementary file 1 [file pharmaceuticals-15-01505-s001.zip › Table S1.pdf]

Table S1 The peak area integration of biflavonoid from SD-BFRE by UPLC-PDA

No.	t_R *(min)*	Molecular iron(m/z)	Area µV*Sec	Area%	Compound
1	12.81	537.0807	28044707	37.99	Amentoflavone
2	13.99	538.0969	1101145	1.49	2,3-Dihydro-3',3''''-biapigenin
3	14.06	541.1167	5545192	7.51	2,3,2'',3''-Tetrahydroochnaflavone
4	15.00	539.1029	6250411	8.47	2，3-dihydroochnaflavone
5	15.21	537.1248	2474981	3.35	Delicaflavone
6	15.22	537.1096	1805727	2.45	Ochnaflavone
7	15.37	537.0983	1366105	1.85	Hinokifllavone
8	16.06	551.0997	1205544	1.63	Bilobetin
9	16.84	565.1285	1820240	2.47	Ginkgetin
10	17.18	551.0983	2717065	3.68	Podocarpusflavone A
11	18.83	579.1281	7505061	10.17	Heveaflavone
12	20.82	593.1569	4040694	5.47	7,4',7'',4'''-Tetra-O-methyl-amentoflavone
