# Supplementary figures and images for: A Biflavonoid-Rich Extract from Selaginella doederleinii Hieron. against Throat Carcinoma via Akt/Bad and IKKβ/NF-κB/COX-2 Pathways
Source: Pharmaceuticals (Basel). 2022 Dec 2;15(12):1505. doi: 10.3390/ph15121505 (PMC9785591; doi:10.3390/ph15121505)

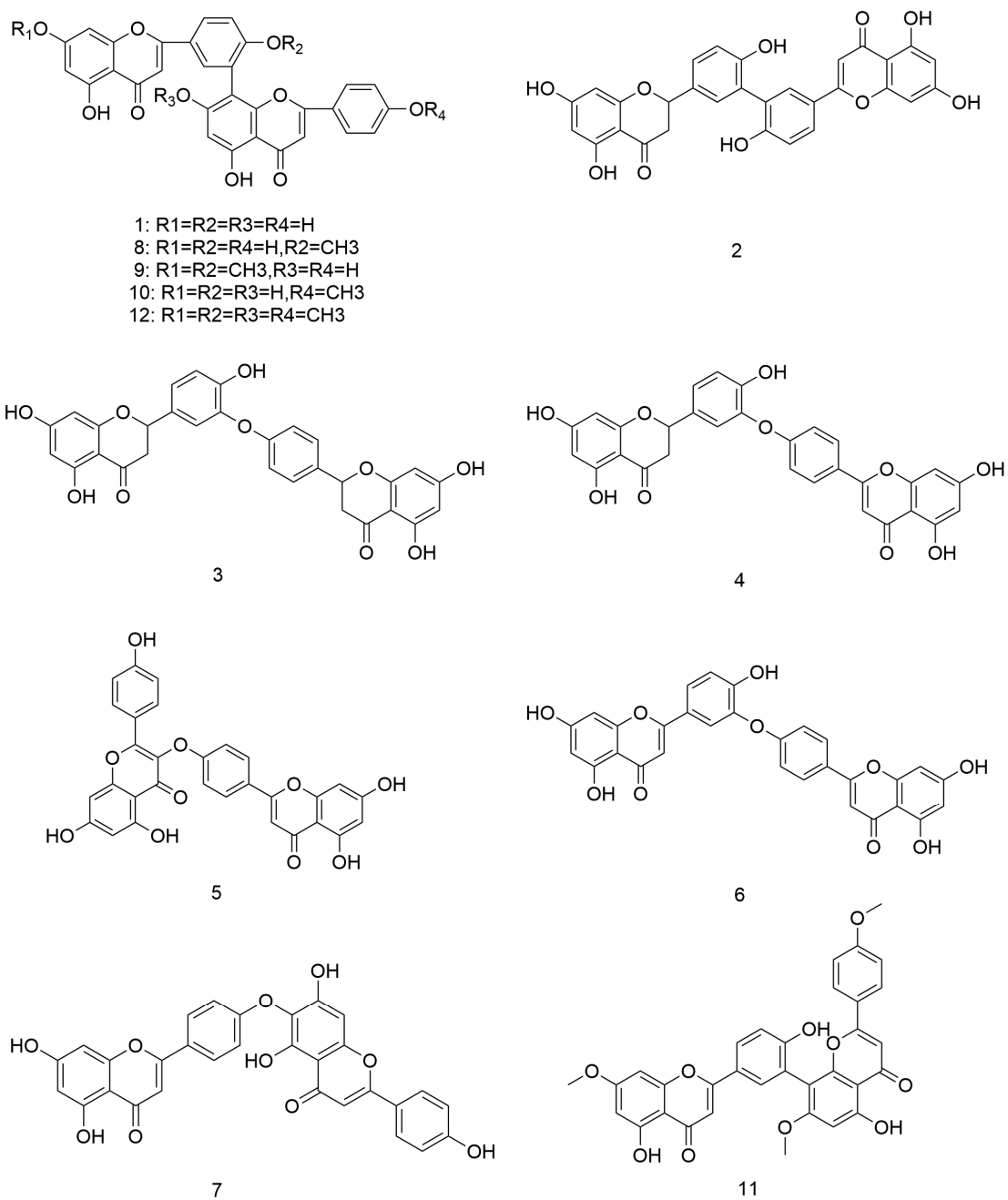

**Figure S2:** The chemical structure of 12 biflavonoids identified from SD-BFRE by UPLC-Q-TOF-MS.

Supplement: Supplementary file 1 [file pharmaceuticals-15-01505-s001.zip › Figure S2.pdf]
